# What do physicians think about the white coat, about patients' view of the white coat, and how empathetic are physicians toward patients in hospital gowns? An enclothed cognition view

**DOI:** 10.3389/fpsyg.2024.1371105

**Published:** 2024-06-11

**Authors:** Gillie Gabay, Hana Ornoy, Dana O. Deeb

**Affiliations:** ^1^School of Sciences, Achva Academic College, Shikmim, Israel; ^2^School of Business Administration, Ono Academic College, Kiryat Ono, Israel; ^3^Josselson Integrated Heart Center, Shaare Zedek Medical Center, Jerusalem, Israel

**Keywords:** hospital, enclothed cognition, physicians, empathy, the white coat, physicians' view of patients, professional identity, patient hospital gown

## Abstract

The receipt of the white coat by medical students is a significant milestone. Extensive research has focused on the white coat, its purity, representation of authority and professionalism, its role in consolidating a medical hierarchy, and the professional status attributed to physicians wearing it. Studies suggest that the white coat is a symbol of medical competence, and patients expect physicians to wear it. Research, however, has paid little attention to what physicians think about their white coat, how they perceive the patient's view of the white coat and the hospital gown, within the patient–physician power asymmetry, which is the focus of the current study. In total, 85 physicians from three Israeli medical centers completed a questionnaire (62% Muslims, 33% Jewish, and 5% Christians; 68% men, ages ranging from 21 to 73 years). Employing the enclothed cognition theory and adopting a within-person approach, we found that the more physicians perceived the white coat as important, the more they attributed a positive view of the white coat to patients and the more they perceived the patient's view of the hospital gown as positive. In addition, the higher the perceived importance of the white coat, the higher the reported empathy of physicians toward inpatients, which is consistent with the hospital's values of care. Interestingly, although medicine is a symbol of protection and care for others, the symbolic meaning of the white coat was potent enough to elicit empathy *only* when physicians perceived it as important. This study extends the theoretical knowledge on the theory of enclothed cognition in healthcare regarding self-perceptions and professional conduct.

## Introduction

Work uniforms are distinctive to certain professions (Chang and Cortina, 2024). They suppress individual personality and enhance the sense of belonging to a professional group. Studies have shown that uniforms trigger stereotypes about a professional group. Therefore, to promote conformity within their ranks and project a desired image to the outside world, institutions devote considerable resources to establishing, enforcing, and even litigating formal dress codes (Ford, 2022). The physician's whitecoat has been studied more than any other uniform (Chang and Cortina, 2024) and its conferral on medical students is a meaningful milestone. The research highlighted the whitecoat's symbolic function: its connotations of purity, its representation of authority and professionalism, its role in consolidating a medical hierarchy, and the professional status patients attribute to the wearer (Loh et al., 2000; Treakle et al., 2009; Wellbery and Chan, 2013; Gouraud et al., 2014; Palacios-González and Lawrence, 2015).

Physicians wearing white coats were perceived as more authoritative than doctors in suits and ties (Brase and Richmond, 2004). Dentists in white coats were perceived as more capable than dentists in smart or casual attire (Furnham et al., 2013). The white coat has been associated with perceived competence and supernatural powers (Blumhagen, 1979). Thus, it represents the social identity of physicians and triggers the generally positive stereotypes associated with medicine. Older adult patients expected physicians to wear a white coat (Wilson et al., 2007; Crutzen and Adam, 2022). The white coat increased Japanese patients' trust in physicians and satisfaction with care (Kamata et al., 2020). American patients placed higher confidence in physicians adorning a white coat compared to physicians who are differently attired (Rehman et al., 2005). Some suggest that it is morally wrong for physicians to wear other attire, as it diminishes their professional image as competent physicians (Wellbery and Chan, 2013; Palacios-González and Lawrence, 2015).

Scholars have not yet addressed the question of how physicians perceive the white coat and how it impacts their thoughts and perceived self-conduct. This study aimed to explore the meaning that physicians derive from the white coat, its linkage to their perception of the patient's hospital gown, and their perceived conduct, according to the enclothed cognition theory.

## The enclothed cognition theory

To explain how work attire impacts individual cognition, Adam and Galinsky (2012) introduced the concept of “enclothed cognition,” which describes how attire orients the wearer toward the symbolic meanings associated with the clothing worn. Adam and Galinsky (2012) theorized that two factors produce enclothed cognition: (a) the symbolic meaning associated with an item of clothing and (b) the physical experience of wearing that item. The enclothed cognition framework argues that wearing certain clothing causes people to “embody” the symbolic meaning of the clothing and subsequently affects their psychological processes (Adam and Galinsky, 2012). The theory suggests that when a particular item of clothing is worn, it exerts an influence on the wearer's psychological processes by activating associated concepts through its symbolic meaning (Adam and Galinsky, 2012).

Attire may represent issues inherent to professional identity, stemming from the symbolic associations of the professional attire that conveys information about social hierarchy. Thus, different choices of attire can highlight competing demands, such as being professional or being authentic (Bailey et al., 2022). Enclothed cognition theory helps clarify how employees' clothing may impact their self-perception. For example, wearing business suits activates the implicit meaning of personal control, which leads the wearers to assert this control over healthier eating choices (Wang et al., 2021). Thus, by examining the symbolic meaning(s) associated with the clothes that employees wear to work, we can better understand the effects of the clothes on employees' cognition and subsequent behaviors. Enclothed cognition theory refers to the systematic influence that attire may have on the cognition, emotions, and behaviors of the wearer (Lopez-Perez et al., 2016; Horton et al., 2023; Kim et al., 2023; Pech and Caspar, 2023). A study that tested the psychological impact of attire on authenticity, power, and work engagement found that attire activated all these cognitive schemas (Gino and Kouchaki, 2020; Bailey et al., 2022).

Chang and Cortina (2024) extended the theory and introduced the concepts of enclothed harmony and enclothed dissonance. Harmony captures symbolic consistency with the context, whereas dissonance captures inconsistency with the context. The same attire may produce both enclothed harmony and dissonance depending on the context. Because people are averse to dissonance, employees prefer harmony with the work context and organizational values (Ramarajan et al., 2017; Ebrahimi et al., 2019). Enclothed cognition theory has been gaining empirical support. When individuals were assigned to wear formal attire, they described themselves as more determined and rational, whereas when assigned to wear casual attire, they described themselves as more emotional and easygoing (Hannover, 2002). Moreover, respondents who wore a suit (“upper-class clothing”) reported greater dominance relative to those wearing sweatpants (“lower-class clothing”) (Kraus and Mendes, 2014). Furthermore, when workers dressed professionally, they thought more abstractly and used more formal language to describe themselves, as a consequence of feeling powerful (Slepian et al., 2015).

In healthcare, wearing a white lab coat improved concentration when the lab coat was framed to participants as a “medical doctor's coat,” but not when the same white lab coat was described as an “artist's coat” (Adam and Galinsky, 2012). The Red Cross uniform increased neural responses to pain, compared to civilian or military attire (Gino and Kouchaki, 2020). In addition, wearing and identifying with the nurse's uniform affected the vicarious emotions and behaviors of nurses and increased their empathy and altruistic motivations (Lenth, 2006; Adam and Galinsky, 2012; Houweling et al., 2014; Lopez-Perez et al., 2016; Horton et al., 2023; Moody, 2023; Pech and Caspar, 2023).

Furthermore, wearing professional attire increased mental abstraction (Douse et al., 2004), problem-solving (Frankel et al., 2021), and prosocial behaviors in medical students (Adam and Galinsky, 2012; Lopez-Perez et al., 2016). It was observed in a study that medical students who wore a white lab coat displayed higher attentional control toward problem-solving (Adam and Galinsky, 2019).

## Rationale for the current study

Limited studies that examined how uniforms shaped employees' social identities did not include the impact of their identity on behaviors. Moreover, most of the studies employing the theory of enclothed cognition focused on what the clothing communicated to others (Bonaccio et al., 2016; Kim et al., 2023; Chang and Cortina, 2024). People generally associate the physician's coat with scientific focus and attentiveness (Adam and Galinsky, 2012), but how does it impact the physicians' thoughts and attitudes?

Considering the possibility that the white coat is infused with symbolic meaning that may impact the psychological processes of physicians, we aimed to bridge the gap between practice and scholarly interest. We also aimed to understand whether the white coat matters to physicians and whether its symbolic meaning affects them, thereby extending enclothed cognition theory to physicians (Adam and Galinsky, 2012). The goals of this study were as follows: (a) to employ enclothed cognition theory in testing how the white coat holds symbolic meaning and impacts physicians' perceptions; (b) in contrast to previous research (Rafaeli and Pratt, 1993), to adopt a within-person perspective in studying the enclothed cognition of physicians wearing the white coat; (c) to explore whether the influence of the white coat on the self-perceptions of physicians is linked to their perceptions about the clothing of patients; and (d) to understand how the white coat gauges the physicians' relational attitude toward hospitalized patients.

## Developing hypotheses

Applying the theory of enclothed cognition to physicians, we propose that aspects of the white coat as symbolically meaningful will impact the self-perceptions and subsequent relational behaviors of physicians (Lopez-Perez et al., 2016; Horton et al., 2023). Following the enclothed cognition theory, since cognitive functions are not confined to the brain but are also shaped by the mutual interactions between the brain, the body, and the external environment (Pech and Caspar, 2023), we hope, according to enclothed cognition theory (Adam and Galinsky, 2012), that wearing the white coat will activate its symbolic meanings. We argue that the symbolic meaning of the white coat can represent concepts that extend beyond its functional use and will pervade psychological processes (Adam and Galinsky, 2012). Based on the above review, we expect physicians who identify with the white coat to have a positive view of it and assume that patients also view it positively. We, therefore, hypothesize that physicians will perceive the white coat as being associated with greater attentiveness to patients.

## Conducting the study

After receiving ethical approval from the ethical board of the academic institution with which the first author is affiliated (IRB#117), the questionnaire in English was translated into Hebrew and Arabic by a certified translator affiliated with the Israel Translators Association. The study was presented to physicians at morning staff meetings. Participation was voluntary, and participants signed an informed consent for participation and publication. Participants who agreed to participate filled out the questionnaires at the end of staff meetings in the respective hospitals. The distribution and collection process spanned ~3 months.

Participants were physicians from three Israeli hospitals (*n* = 85), with 68.2% of them being men and 31.8% women with ages ranging from 22% to 74 years (M = 37; SD = 11), from diverse religions and religiosity (62.4% Muslims, 32.9 Jewish, and 4.7 Christians; 39% defined themselves as religious, 25% as traditional, 24% as secular, 9% as partially traditional, and 4% as very religious). When asked whether physicians wearing a white coat have more empathy for patients, 45% completely disagreed, 25% moderately disagreed, 15% disagreed, 14% agreed, and 1% completely agreed. Table 1 presents the mean and distribution of the perceived importance of the white coat (α = 0.735), the perceived view of the hospital gown among patients (α = 0.91), and the perceived view of the whitecoat among patients (α = 0.949). Table 2 presents the correlations among study variables.

**Table 1:** Mean and distribution of the white coat and the patient gown as perceived by physicians.

**Variable**	**Mean**	**SD**	**Minimum**	**Maximum**
Physicians' perception of the importance of the white coat	2.65	0.76	1	4.57
Perception of the physician of how positively the patient perceives his gown	2.4	1.06	1	4.8
Physicians' perceived positive view of the white coat by patients	2.83	1.16	1	5

**Table 2:** Pearson and spearman correlations between the variables.

	**Physicians' perception of the patients' view on the hospital gown**	**Physicians' perceived empathy toward patients**	**Physicians' perception of the patient's view on the white coat**
Physicians' perceived importance of the white coat	*r*_*p*_ = 0.546 *p* < 0.001	*r*_*s*_ = 0.376 *p* < 0.001	*r*_*p*_ = 0.397 *p* < 0.001

A significant positive relationship was found between the physician's perceived importance of the white coat and the perceived patient view of the patient's hospital gown. Furthermore, a significant positive correlation was found between the physicians' perceived importance of the white coat and physicians' empathy toward hospitalized patients. Moreover, a significant positive correlation was found between the physicians' perceived importance of the white coat and their perception of patients' positive view of wearing a white coat. In addition, the perceived importance of the white coat in physicians differed by religion. While 26% of Jewish and 25% of Muslim physicians rated the importance of the white coat as 1–3, 38% of Muslims and 32% of Jewish physicians rated the importance as 3–4. Furthermore, 4% of Muslims vs. 11% of Jewish physicians rated the importance of the white coat as 4–5.

## Discussion

The study tested the enclothed cognition theory regarding the professional attire of physicians. Consistent with the enclothed cognition theory, the relationship between the white coat and feelings of empathy created a harmonic cognitive identity, congruent with the values of care at the hospital. This alignment may reflect a higher engagement of physicians with work (Glavas, 2016; Sutton, 2020). Building upon the enclothed cognition theory, we found a significant positive relationship between the physicians' perceived importance of the white coat and the physician's perception of how patients view their hospital gowns. The more physicians perceived the white coat as important, the more they perceived the patient gown as important as well. We found a significant positive correlation between the physicians' perceived importance of the white coat and their perceived positive view of the white coat among patients.

Finally, the perception of the white coat among physicians was associated with higher self-reported empathy toward patients. Interestingly, although medicine is a symbol of protection and care for others, the symbolic meaning of the white coat was potent enough to elicit empathy only when the white coat was perceived by its wearers as important (Van Stockum and DeCaro, 2014; Goldinger et al., 2016; Pfattheicher et al., 2022).

Echoing a study with nurses, our results indicate the extent to which the white coat is associated with caring and prosocial concepts, and with physicians' compassionate responses to a patient's suffering, supporting the enclothed cognition theory (Wołoszyn and Hohol, 2017; Zwaan, 2021). This finding aligns with the previous studies that have demonstrated a decline in empathy toward patients over time among medical students not wearing white coats (Hein and Singer, 2010; Gallo et al., 2018). Diminished empathy may be explained by the habituation effect or by the physician's need to regulate their empathy as a cognitive resource, to protect themselves from distress and compassion fatigue as they witness the suffering of hospitalized patients (Decety et al., 2010; Smith et al., 2017).

## Contributions of the study

Our study applies the enclothed cognition theory in the hospital context and integrates cognitive psychology, social psychology, and organizational behavior within healthcare, linking professional uniforms to organizational outcomes.

It makes several theoretical contributions. First, building upon the findings of the enclothed cognition theory (Adam and Galinsky, 2019) offers a general theory of the effects of the white coat on physicians wearing it, conveying values of care in the hospitalization context. We demonstrated that the psychological effect of wearing the white coat extends beyond its functional use and universal meaning, to the relational value of empathy toward hospitalized patients. It is highly notable that our study makes an important contribution by highlighting the choice to wear the white coat as affecting self-perceptions and behavior at the hospital.

Although hospitals make increasing efforts to shape the relational behaviors of physicians toward patients, we show that the white coat can contribute to that goal within the hospital context. Our findings demonstrate that the choice of wearing the white coat matters, contributing to the psychological literature, which, thus far, largely adopted a between-organization and an organization–customer approach. In drawing from the enclothed cognition theory (Adam and Galinsky, 2012), our study contributes to that line of theorizing. Although initial theorizing did not preclude the possibility that fluctuations in physicians' attire may affect the cognition and behavior of the physician, empirical research, thus far, mainly focused on specific clothing with clear and universal symbolic meanings (Lopez-Perez et al., 2016; Adam and Galinsky, 2019; Wang et al., 2021).

We provided evidence for the nature of the symbolic meaning of the white coat and showed its perceived importance in the perceived view of the patient and in empathy toward patients wearing the hospital gown. Thus, the white coat expressed the identity of physicians at the hospital signaling a fit with their social work environment (Roach-Higgins and Eicher, 1992). We revealed that the psychological outcome of empathy is associated with wearing a white coat. Contrary to previous studies, the white coat did not signal an increased sense of power toward hospitalized patients (Rafaeli et al., 1997). In support of a previous study, physicians wearing white coats were more engaged in expressing empathy toward hospitalized patients, reflecting an identity consistent with the work context (Bailey et al., 2022).

Our understanding of the impact of the white coat within the hospital setting and the context of patient care is that attire and work context are aligned (Rafaeli et al., 1997). This alignment increased engagement and activated the symbolic meaning of the white coat. We demonstrated that the white coat affects physicians' thoughts and behaviors, thereby extending the enclothed cognition theory to healthcare, supporting the generalizability of this concept beyond the boundaries that were set in the previous studies. Our research unveils a key mechanism through which the physician's clothing at the hospital influences their attitude toward patients.

To date, research employing the enclothed cognition theory indirectly probed potential psychological mechanisms, drawing on the general notion that the symbolic meaning of the clothing one wears becomes embodied by the wearer. Our findings shed light on the specific symbolic meaning of the white coat, revealing the effects of enclothed cognition in hospitals. Furthermore, the within-person approach we adopted adds to the understanding of the enclothed cognition theory, similar to previous research, (Bailey et al., 2022) which focused on enclothed cognition effects at the between-person level. Since theories often operate differently across levels of analysis (Vogel et al., 2020), the within-person lens supports the concept that enclothed cognition effects in the workplace occur within-person and may be applied to improve patient experience in hospitalizations.

Muslims comprise 22% of the Israeli population, however, Muslim physicians comprised 30% of the medical workforce (Lenth, 2006; Taub Report Physicians in Israel Trends in Characteristics and Training, 2024) and 66% of the study sample, supporting the inclusion and difference approach in medicine (Epstein, 2010). Increasing the ethnic diversity of the medical workforce is commonly accepted as a promising means of providing culturally and linguistically competent care to improve the health of minority populations and reduce health disparities (Popper-Giveon et al., 2015). Muslim physicians were more empathetic toward patients wearing the hospital gown, perhaps reflecting the perceptions of medical practice among Muslim physicians as being impartial, universal, and humanitarian, guaranteeing neutral, impartial, and humanitarian healthcare, enabling the inclusion of minority professionals in health organizations (Keshet and Popper-Giveon, 2017).

## Practical implications

The current findings have important practical implications suggesting that to promote engagement with values of care, physicians may be encouraged to wear the white coat. One practical implication of the enclothed harmony perspective is to wear the whitecoat as it is consistent with the work setting and professional values. Physicians have choices regarding whether or not to wear the white coat, which may seem contradictory in terms of fitting in or standing out among peers. However, based on the enclothed cognition theory, the white coat should be considered to impact psychological processes such as one's sense of relational value. Our study suggests a potential downside to the trend away from the white coat as a dress code, a trend that may trigger a negative relational value.

Thus, although physicians may desire greater self-expression at the hospital, management may subtly guide physicians toward making decisions that will positively impact their work behavior. As we argue that the meaning physicians ascribed to their white coats may influence them, the attire may amplify the enclothed cognition processes. This is not to suggest that the white coat represents the foremost means of fostering relational attitudes toward patients, but that the choice of what physicians wear at the hospital may enhance such behaviors. Although the white coat may seem insignificant for some physicians, this study suggests that it is not only symbolically meaningful but also increases consistent cognition identity and enhances empathy toward patients.

## Limitations

The study was conducted exclusively among Israeli physicians, potentially rendering its findings non-representative of perceptions of physicians in other countries. The second limitation is that the sample is not representative of the demographical characteristics of the Israeli medical workforce perceived, perhaps presenting a method bias. The third limitation is by asking participants about the impact of the white coat, they may have inferred the study's objective and attempted to please the researchers by conforming to the expected behavior. The fourth limitation is that we did not inquire about participants' prior experience of wearing the white coat, which could have influenced the outcomes. It is possible that participants were not fully aware of the impact of the white coat on their behavior and cognition. It is likely that certain physicians are more sensitive than others to its symbolic meaning. Although there is evidence to challenge the enclothed cognition framework, caution is required as it is a non-falsifiable theory much like connectivism or cybernetics (Adam and Galinsky, 2012, 2019; Burger and Bless, 2017). Finally, the sample size limited the comparison across religions and age groups.

## Future studies

This research opens new avenues for scholars to understand more about how physicians' choice to wear the white coat influences them and their attitude toward patients. The study calls upon medical educators to stress the importance of the white coat to promote empathy toward hospitalized patients. Future studies are called upon to test the conditions under which the white-coat impact is likely to be strong and test interaction among the physicians' attributes, patient attributes, and contextual factors. Gender differences should also be explored. Future studies may also test cultural differences, as the formality level across cultures may affect perceptions and conduct (Hofstede, 2011). Future studies may investigate the effect of the white coat and its interplay with cognitive effects, comparing physicians wearing it with those who do not, across genders, age groups, and religions and specifically ask participants to what extent they act in accordance with the attire they wear each day.

## Data availability statement

The original contributions presented in the study are included in the article/supplementary material, further inquiries can be directed to the corresponding author.

## Ethics statement

The studies involving humans were approved by Achva Academic College #IRB 117. The studies were conducted in accordance with the local legislation and institutional requirements. The participants provided their written informed consent to participate in this study.

## Author contributions

GG: Conceptualization, Project administration, Supervision, Writing – original draft, Writing – review & editing, Formal analysis, Methodology. HO: Conceptualization, Writing – original draft, Writing – review & editing, Supervision, Data curation. DD: Writing – review & editing, Data curation.
